# *Penicillium nalgiovense* Laxa isolated from Antarctica is a new source of the antifungal metabolite amphotericin B

**DOI:** 10.1186/s40694-014-0011-x

**Published:** 2015-01-17

**Authors:** K Stefan Svahn, Erja Chryssanthou, Björn Olsen, Lars Bohlin, Ulf Göransson

**Affiliations:** 1 Department of Medicinal Chemistry, Uppsala University, Uppsala, Sweden; 2 Department of Clinical Microbiology, Karolinska University Hospital & Karolinska Institute, Stockholm, Sweden; 3 Department of Medical Sciences and Zoonosis Science Centre IMBIM, Uppsala University, Uppsala, Sweden

**Keywords:** Amphotericin B, *Penicillium nalgiovense* Laxa, Antarctica

## Abstract

**Background:**

The need for new antibiotic drugs increases as pathogenic microorganisms continue to develop resistance against current antibiotics. We obtained samples from Antarctica as part of a search for new antimicrobial metabolites derived from filamentous fungi. This terrestrial environment near the South Pole is hostile and extreme due to a sparsely populated food web, low temperatures, and insufficient liquid water availability. We hypothesize that this environment could cause the development of fungal defense or survival mechanisms not found elsewhere.

**Results:**

We isolated a strain of *Penicillium nalgiovense* Laxa from a soil sample obtained from an abandoned penguin’s nest. Amphotericin B was the only metabolite secreted from *Penicillium nalgiovense* Laxa with noticeable antimicrobial activity, with minimum inhibitory concentration of 0.125 μg/mL against *Candida albicans*. This is the first time that amphotericin B has been isolated from an organism other than the bacterium *Streptomyces nodosus*. In terms of amphotericin B production, cultures on solid medium proved to be a more reliable and favorable choice compared to liquid medium.

**Conclusions:**

These results encourage further investigation of the many unexplored sampling sites characterized by extreme conditions, and confirm filamentous fungi as potential sources of metabolites with antimicrobial activity.

## Background

The lack of efficient antibiotics combined with the increased spread of antibiotic-resistance genes characterize a lingering global health problem that must be solved [1-3]. Warnings about this problem have been frequent and consistent, and forecasts of new resistance mechanisms resulting in multiple-drug-resistant microorganisms are being fulfilled [4,5].

Natural products are an important source of novel antibiotics in modern medicine, and have made an unquestionable impact on global health so far [6-8]. Terrestrial microbes are the sources of most known antimicrobial compounds, but there has been a noticeable regression in the discovery of such compounds since the 1980s [9]. This negative trend is disquieting, and highlights the need for improving existing sampling and screening methods of filamentous fungi so as to advance the search for new antimicrobial compounds [10].

Fungi have successfully survived for millions of years under extraordinary circumstances and in varied and challenging environments consisting of extremes in pH, sunlight, temperature, nutrients etc. These organisms have even survived man-made environmental impacts such as heavy metal pollution, antibiotics, and other pharmaceutically active substances. Such exposures qualify fungi as potential sources of metabolites with differing biological activities [11].

Fungi such as *Penicillium rubens* and *Cephalosporium acromonium*, which produce penicillin and cephalosporin, respectively, are prominent examples of microorganisms used in drug discovery. Furthermore, several *Trichoderma* spp. have successfully been used as fungal biocontrol agents instead of synthetic fertilizers and pesticides [12]. The estimated 100,000 species of fungi that are known today make up only about 10 percent of all fungal species that are predicted to exist, implying that fungi are an unmapped and untapped source of secondary metabolites with bioactive potential [13].

Unknown fungal species and their constituent metabolites can be identified by investigating the microflora of soil or biological samples from extreme or unexplored environments. Ideally, the samples will host new microbial species or novel compounds with desired activity [14,15]. Antarctica exemplifies the environments described above, with some of the most extreme terrestrial conditions including low temperatures, high ice coverage (>99%), a sparsely-populated food web, insufficient liquid water availability, high water and airborne salinity, and intense UV-radiation. These conditions have led to the conclusion that microbes endure hostile surroundings in Antarctica, and although recent studies indicate that this might not be the case for bacteria, it is more likely to be true for fungi [16-20]. Limited amounts of carbon and nitrogen substrates inhibit fungal growth in Antarctica, even though debris from last century’s polar expeditions have somewhat improved fungal life conditions and diversified fungal flora [21].

Nonetheless, natural selection excludes any fungi that does not adapt to the harsh environment. This raises the question of whether new secondary metabolites with antimicrobial activity are developed during evolutionary competition between microbes. Earlier investigations of soil samples from Antarctica demonstrated diversity more often in the microbes than in their secreted metabolites [22].

In the current work, we investigated the fungal flora found in the extreme and rarely studied environment in Antarctica with the aim to discover new secondary metabolites with antimicrobial activity.

## Results

### Collection, cultivation and identification of *Penicillium nalgiovense* Laxa

During an expedition to Antarctica in 2009, a soil sample was collected from a penguin’s nest on Paulete Island and transported in an unbroken freeze chain to our laboratory in Sweden. The soil sample harbored a large number of fungi, and one strain was chosen for further analysis due to its strong antimicrobial activity in the initial assays. This strain was identified at the Fungal Cultivation Centre in the Netherlands as *Penicillium nalgiovense* Laxa based on both genotypic characters, morphology and colony patterns. To rule out the possibility of bacterial contamination, DNA was isolated from the cultures of *Penicillium nalgiovense* Laxa and used for PCR with universal 16S rDNA primers. No amplicons indicating bacterial contamination could be detected.

### Extraction and isolation of antimicrobial compound

**Figure 1:**
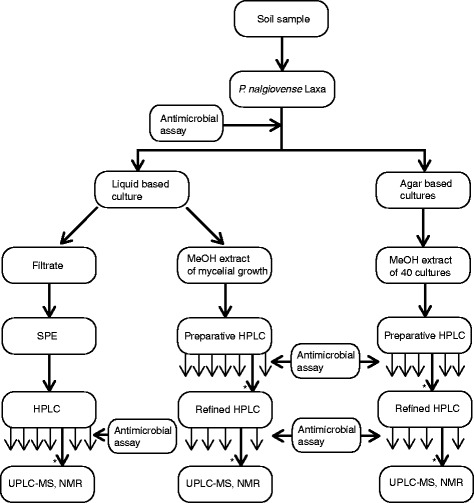
**Extraction procedure.** Cultivation, extraction, and isolation of metabolites with antimicrobial activity from *Penicillium nalgiovense* Laxa. Fractions without antimicrobial activity are marked with thinner arrows under the HPLC boxes, and those with antimicrobial activity are marked with an asterisk (*).

The extraction of fungal cultures aimed to identify all secreted compounds with antimicrobial activity. Three major extracts were analyzed: the filtrate of the liquid culture, the mycelial growth in the liquid culture, and the cultures growing on solid medium (Figure 1).

**Figure 2:**
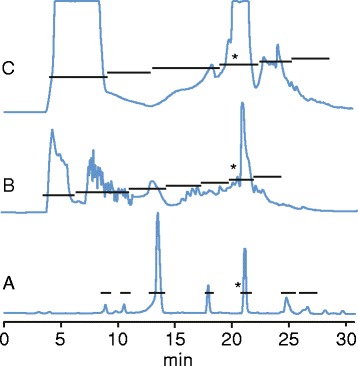
**Preparative work of fungal extracts.** Chromatograms from preparative isolation recorded at 280 nm of **(A)** filtrate of liquid-based fungal culture, **(B)** methanol extracts of mycelial growth, and **(C)** methanol extract of fungal cultures on solid medium. The horizontal bars indicate fractionation, and the asterisks (*) indicate antimicrobial activity. Amphotericin B eluted at 21.50 min.

The filtrate of the liquid culture contained sugars and salts (potassium chloride, sodium chloride, magnesium sulfate etc.), which originated from the RPMI 1640 medium. These contents were separated from the filtrate with reverse phase solid phase extraction (SPE). The SPE fraction was freeze dried, dissolved in 10% acetonitrile with 0.05% trifluoroacetic acid (TFA), and fractionated with preparative reverse phase high performance liquid chromatography (HPLC), yielding seven fractions (Figure 2A). The fourth fraction included one peak with yellow color eluting at 21.50 minutes.

The methanol (MeOH) extract from the mycelial growth was evaporated, dissolved in 10% acetonitrile with 0.05% TFA, and fractionated with HPLC, yielding seven fractions (Figure 2B). The sixth fraction was yellow with a large peak at 21.45 min, but mass spectrometry (MS) analysis showed that it contained several substances. To separate its contents, this fraction was freeze dried, dissolved in 30% acetonitrile with 0.05% TFA, re-injected into an analytical HPLC column, and fractionated with a more gradual gradient.

The MeOH extract from the solid medium cultures had a yellow-orange color, and was fractionated with HPLC as described above. The fourth fraction, which originated from one large broad peak emerging at about 21 min was yellow (Figure 2C), contained several substances and was therefore fractionated again and analyzed in the same manner as the mycelial growth extract.

### Antimicrobial assays

All fractions and sub fractions were tested for antimicrobial activity (Figure 1). Fractions 5, 6, and 4 (Figure 2) from the filtrate, mycelial growth extract, and solid culture extract, respectively, all had a yellow color and showed antifungal activity (MIC value 0.125 μg/mL against *Candida albicans*) and weak antibacterial activity (4.0 μg/mL against *Escherichia coli* and *Staphylococcus aureus*).

### Identification and yield of amphotericin B

**Figure 3:**
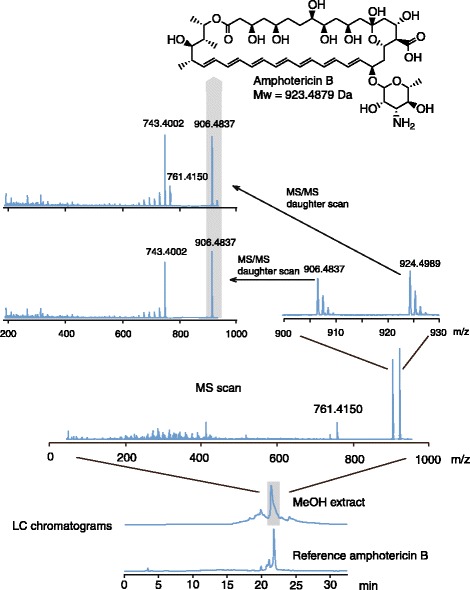
**Identification of amphotericin B.** The preparative and analytical results of the experiments identifying the only substance with antimicrobial activity in a culture extract of *Penicillium nalgiovense* Laxa. The methanol extracts of the mycelial growth and liquid filtrate were fractionated with HPLC (bottom two chromatograms recorded at 280 nm), analyzed with Q-TOF MS (middle spectra), and compared with the reference substance amphotericin B (top figure). Identical elution times (21 minutes 50 seconds), mother ions (924.4989 and 906.4837 m/z), and daughter ions (743.5, 761.5 m/z) between the extracted compound and reference amphotericin B, together with NMR analysis and antimicrobial assays, led to the identification of amphotericin B in the fungal extracts.

**Figure 4:**
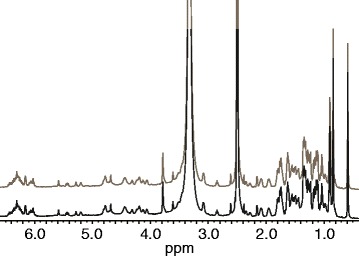
^**1**^ **H NMR spectra of extract (below) and reference amphotericin B (above) dissolved in dimethylsulfoxide-** ***d6*** **.**

Reference amphotericin B was tested in the antimicrobial assay and analyzed with HPLC, MS (Figure 3), and nuclear magnetic resonance (NMR) spectroscopy (Figure 4) to help identify the isolated compound. Reference amphotericin B, in the form of an orange powder that turned yellow in solution, had an identical retention time (21.50 min) compared to the active substances in the extracts, and produced MIC values of 0.250 μg/mL against *Candida albicans* and 4.0 μg/mL against *Escherichia coli* and *Staphylococcus aureus*. The weights of the fractions with extracted amphotericin B were 0.1, 0.2, and 1.2 mg from the filtrate of the liquid culture, the mycelial growth, and the solid phase, respectively. Thus, the concentration of amphotericin B in the 1250 mL filtrate was 80 ng/mL, and 4 μg/mL in the 50 mL mycelial growth.

The three active fractions each had a cluster of ions eluting at the same time on the ultra high performance liquid chromatography (UPLC) system (36 min 40 sec, as seen in reference amphotericin B) with mass-charge (m/z) ratios of 924.4989, 906.4837, and 761.4150 (Figure 3; reference amphotericin B: 924.4897 m/z, calc. 924.4952). The first two ions were (M + H)^+^ and (M + H-H_2_O)^+^, respectively, and the third, (M + H-C_6_H_12_NO_4_)^+^_,_ corresponds to the loss of the mycosamine ring. The ions with m/z ratios of 924.5 and 906.5 were analyzed with tandem MS and generated daughter ions mainly between 650 and 762 m/z (Figure 3). Except for ions with 761.5 m/z, the isolated compounds had an identical set of daughter ions, indicating that the parent ions with m/z ratios of 924.5 and 906.5 m/z ions originate from the same metabolite. Reference amphotericin B had identical spectra. Tandem MS experiments with collision energies up to 80 V were performed to detect ions with a lower m/z than 906.5, but did not reveal more information than that already presented in Figure 3. To exclude possible chemical contamination, all growth media were screened for amphotericin B. No amphotericin B was found.

The active fractions of amphotericin B from all three extracts (total weight 1.5 mg) were dissolved in dimethylsulfoxide-*d6*. The ^1^H spectra of the extract and reference amphotericin B (Figure 4) were identical and comparable to available NMR data from amphotericin B [23,24].

## Discussion

We have isolated the antifungal secondary metabolite amphotericin B from *Penicillium nalgiovense* Laxa found in soil from a penguin’s nest in Antarctica. Isolated amphotericin B was identified by comparing the solution color, antimicrobial activity, and chromatographic, spectrometric and spectroscopic data to those of a reference sample of amphotericin B.

Earlier studies of *Penicillium nalgiovense* Laxa have resulted in the isolation of e.g. penicillins, diaporthins, nalgiovensin, and dipodazin [25-28]. Why amphotericin B was not extracted from *Penicillium nalgiovense* Laxa sooner when its existence has been acknowledged for more than 80 years is unknown, but neither of earlier studies has used our specific cultivation, extraction and isolation approaches. In those previous studies, the choice of growth media included e.g. moistened wheat bran, the glucose based Raulin-Thom solution, Czapek yeast autolysate agar, malt extract agar, and oatmeal agar. To our knowledge, the current work is the first time potato dextrose agar with coconut flakes is used for cultivation of *Penicillium nalgiovense* Laxa. Previous extraction solvents include petroleum, chloroform-methanol mixtures, and ethyl acetate. Although amphotericin B is soluble or slightly soluble in these solvents, none of these studies have followed the antimicrobial activity of the extracts. Hence, our choice of medium in combination with an assay selecting for metabolites with antimicrobial activity likely explains why those earlier studies failed to detect amphotericin B.

Similarly, the same reasons could explain why we did not detect the other *Penicillium nalgiovense* Laxa metabolites mentioned above in the current work. In addition, comparing the results from those earlier studies demonstrate that the variation of expressions seems highly variable as none of them report the same set of secondary metabolites [25-28]. Together with the results in the current work, it is clear that not all strains of *Penicillium nalgiovense* Laxa produce and secrete identical sets of metabolites, and discrepancies in fungal metabolism could be expected.

Our cultivation procedure was noticeably improved by using potato dextrose agar enriched with coconut flakes instead of the liquid RPMI 1640 medium. The fungus did usually not produce amphotericin B in liquid medium, or sometimes no metabolites at all. In successful experiments, the highest concentration of amphotericin B was found in the 50 mL mycelial growth in the liquid culture at a concentration of 4 μg/mL to a total mass of 0.2 mg. Cultures based on solid media generated an overall better yield, as we repeatedly managed to extract amphotericin B from the first cultivation attempt and in larger amounts during a shorter period of time. A culture the size of 40 petri dishes (24,5 dm^2^) rendered a total mass of 1.2 mg amphotericin B, thus this method was consider more favorable, since it was cheaper, quicker, and more reliable in terms of metabolic production.

Amphotericin B is an antifungal agent used worldwide against fungal infections since 1959 [29]. It is prescribed against infections caused by microbes including *Candida* spp., *Aspergillus* spp., *Leishmania* spp., [30,31] and zygomycetes, but is not used against bacteria, since it has no, or limited, antibacterial activity [32]. It was discovered in 1955 when isolated from a strain of *Streptomyces nodosus* found in a soil sample from the Orinoco River in northern South America. The activity of amphotericin B is caused by its affinity to ergosterol, which is a principal sterol in fungal cell membranes. Amphotericin B acts by binding to ergosterol and creating a pore, with the polyene side oriented towards the membrane [33]. Through this pore, ions and small molecules can leak in and out of the cell, which causes metabolic disruption and eventually cell death [34,35]. The extracts in this study showed activity against *Candida albicans*, which is expected of amphotericin B, but also demonstrated weak antibacterial activity against *Escherichia coli* and *Staphylococcus aureus*. This is unexpected since ergosterol, to which amphotericin B binds, is present in neither bacterial cell walls nor membranes [32].

So far, there are no reports of other organisms, except *Streptomyces nodosus*, producing amphotericin B [36,37]. Amphotericin B is biosynthesized in *Streptomyces nodosus* by the genes *amphA-C, amphDI-III, amphG-N*, and three open reading frame genes [29]. Although this study did not investigate the biosynthetic mechanism for the production of amphotericin B in *Penicillium nalgiovense* Laxa, it could be explained by a lateral gene transfer from *Streptomyces nodosus* or another organism able to produce amphotericin B. If not, a different biosynthetic mechanism may have been developed independently by either this strain of *Penicillium nalgiovense* Laxa or by the genus itself. It can also be argued that dormant genes in this organism need specific activation cues to produce secondary metabolites with or without antimicrobial activity. Since the genes of *Penicillium nalgiovense* Laxa are still not mapped, this remains unclear and is considered a field of future studies.

The filamentous fungus *Penicillium nalgiovense* Laxa was discovered and classified in 1932 by O. Laxa and is used today in the food industry as a starting culture for sausages, generating their white-greyish colored cover [27]. To date, with the exception of penicillin and some peptaibols (peptides consisting of five to 20 amino acids, including the unusual amino acid alpha-aminoisobutyric acid, and a C-terminal alcohol), there are no reports of antimicrobial metabolites being produced by *Penicillium nalgiovense* Laxa, thus making it suitable for the food industry [38]. Even the most common fungal toxins are not produced by *Penicillium nalgiovense* Laxa, except in a few cases for some strains [25,26,39,40]. A fungus cultured on a sausage might not necessarily produce the same set of metabolites as it would in a laboratory, but our results show that the current use of *Penicillium nalgiovense* Laxa in food production may be taken under consideration, since amphotericin has activity against eukaryotic organisms.

Fungi have a history of being rich in production of secondary metabolites of various types with a wide range bioactivity, which have been the source of a number of pharmaceutically useful drugs [41]. In the search for metabolites with novel structures and activity, diverse terrestrial and marine environments have continuously being screened [8,42]. One of the least explored sites in this regard is Antarctica. This continent offers a hostile environment for any organism with e.g. low temperature, sparsely distributed water, and limited food web, and is relatively difficult to approach in the screening for bioactive compounds originating from microorganisms. Collections on this location are expensive and cumbersome compared to almost any other and studies of the micro flora and bioactive compounds have so far been few [22]. This is the first time *Penicillium nalgiovense* Laxa have been isolated from Antarctica.

Even though this fungus species belongs to as well-studied a genus as *Penicillium*, and has been known and cultivated for more than 80 years, investigational approaches that involve searching new environments for new antibiotic metabolites demonstrate that known fungi still have unmapped metabolism and deserve more in-depth studies.

## Conclusions

This study has shown that a strain of *Penicillium nalgiovense* Laxa can produce the antifungal metabolite amphotericin B, which was more favorably produced when the fungus was cultivated on solid, rather than in liquid media. The fungus was isolated from a soil sample found in a penguin’s nest in Antarctica, which provides an extreme and hostile environment. *Penicillium nalgiovense* Laxa is the second organism, after *Streptomyces nodosus*, reported to produce this metabolite. This work demonstrates the value of natural product extraction from filamentous fungi, even from a well-studied genus like *Penicillium*, and suggests further investigation of unexplored sampling sites characterized by extreme conditions.

## Methods

### Chemicals

All water was filtered through an A10 0.22 μm Millipore filter (Merck Millipore, Billerica, MA), unless otherwise stated. Extraction and chromatography was performed using acetonitrile (VWR, Radnor, PE), trifluoroacetic acid (IRIS Biotech, Marktredwitz, GER), and MeOH (Merck, Darmstadt, GER). The MS analysis was carried out with water, formic acid, and acetonitrile (Sigma Aldrich, St Louis, MO), and NMR spectroscopy with dimethylsulfoxide-*d6* (Armar Chemicals, Döttingen, SWI). Reference amphotericin B was purchased from Abcam (Cambridge, UK).

### Fungal collection, cultivation, identification, and PCR analysis

A soil sample from a recently abandoned Adélie Penguin (*Pygoscelis adeliae*) nest located on Paulete Island (63° 35′ 0″ S, 55° 47′ 0″ W) on Antarctica was collected in a tube containing cryo-protective broth. The sample was frozen at -70°C and transported in an unbroken freeze chain to the Department of Clinical Microbiology at Karolinska University Laboratory in Stockholm, Sweden. The sample was cultured onto Sabouraud dextrose agar (Oxoid, Hampshire, UK), which was incubated at room temperature until colonies were visible. A large number of molds were isolated from the sample. The *Penicillium* sp. strain, which showed the strongest antibiotic activity in initial assays, was chosen for this study.

The strain was identified as *Penicillium nalgiovense* Laxa at the Fungal Biodiversity Center in the Netherlands. The fungus was cultured on malt extract agar, oatmeal agar, synthetic nutrient agar and dichloran 18% glycerol agar. DNA was extracted from the malt extract agar plate after an incubation period of 3 days in the dark at 25°C using the MoBio - UltraClean Microbial DNA Isolation Kit. Fragments containing of part of the alpha-tubulin gene was performed using the primers Bt2a (GGTAACCAAATCGGTGCTGCTTTC) and Bt2b (ACCCTCAGTGTAGTGACCCTTGGC) [43]. The PCR fragments were sequenced with the ABI Prisma Big DyeTM Terminator v. 3.0 Ready Reaction Cycle sequencing Kit. Samples were analyzed on an ABI PRISM 3700 Genetic Analyzer and contigs were assembled using the forward and reverse sequences with the program SeqMan from the LaserGene package. The sequences were compared on GenBank using BLAST and in a large fungal database of CBS-KNAW Fungal Biodiversity Centre with sequences of most of the type strains. The results had a 100% match in all databases with the type strain of *Penicillium nalgiovense* Laxa.

PCR analysis at Uppsala University was performed with a High Pure PCR Template Preparation Kit version 19 (Roche, Basel, CH) according to manufacturer’s instructions. Regions within 16S rDNA genes were amplified by PCR using purified nucleic acids as a template, and primers (5′ - 3′) E341F: CCTACGGGNGGCNGCA and E1406R: GACGGGCGGTGWGTRCA [44]. The PCR products were analyzed using a MultiNA (Shimadzu, Kyoto, JPN) according to manufacturer’s instructions.

An Eppendorf Mastercycler (Eppendorf, Hamburg, GER) was used for 16S rDNA amplification with the following PCR conditions: initial denaturing step: 95°C for 5 min; amplification: 40 cycles of denaturation at 94°C for 30 sec, annealing at 59°C for 30 sec and elongation at 74°C for 1 min; final elongation step: 74°C for 10 min.

Two different attempts to cultivate the isolated fungus *Penicillium nalgiovense* Laxa were made: in liquid and on solid growth media. For the first attempt, the conidia collected from Sabouraud dextrose agar were suspended in 1300 mL RPMI 1640 liquid medium (Sigma Aldrich, St Louis, MO) with pH 7.2 to reach a concentration of 4 – 6 × 10^8^ conidia per mL. The culture was divided into 33 Falcon tubes and incubated under static and aerobic conditions at 20°C. After 14 days, the mycelial growth and the liquid medium were separated by paper filtration (Munktell, Falun, SWE). For the second attempt, conidia were dispersed on forty petri dishes (9 cm in diameter corresponding to a total area of 25,4 dm^2^) with potato-dextrose agar (pH 5.6) mixed with coconut flakes to enrich nutrient content [45,46]. The cultures were incubated for seven days at 20°C under static and aerobic conditions. Both cultivation attempts were carried out in the dark and aerobic conditions were ensured with slightly open lids.

### Extraction and preparative HPLC

The filtrates of the liquid cultures were separated with SPE, using C18 columns with 10 g packing material (Biotage, Uppsala, SWE). The columns were conditioned with MeOH for 1 hour and equilibrated with 10% acetonitrile and 0.05% TFA. Sixty mL of filtrate was added to each column, eluted with 40 mL of 80% acetonitrile with 0.05% TFA, evaporated, and dissolved in 80 mL 10% acetonitrile with 0.05% TFA. The mycelial growth residue was subjected to extraction with 100 mL MeOH three times. Metabolites from the forty fungal cultures on solid medium were extracted with 10 mL MeOH each for 10 minutes. The two MeOH extracts were evaporated in a speed vacuum centrifuge (Speed Vac Plus SC110 A, Savant Instrument, NY) and dissolved in 20 mL (mycelial growth extract) or 50 mL (extract from solid medium cultures) 10% acetonitrile with 0.05% TFA.

Preparative isolation was carried out using an ÄKTA Basic 10 HPLC system (Amersham Pharmacia Biotech, Sweden) with a C18 250 × 10 mm, 5 μm Jupiter column (Phenomenex, Torrance, CA). The UV-900 detector operated at 210 nm, 254 nm, and 280 nm, and the P-490 pump was set to 5 mL/min. Eluent A and B were 10% acetonitrile and 60% acetonitrile, respectively, both with 0.05% TFA. After 5 minutes of isocratic 0% eluent B, the gradient was increased to 100% B within 44 minutes. Some fractions that contained several substances needed further preparative work. Those fractions were freeze dried and dissolved in 5 mL 30% acetonitrile with 0.05% TFA, and injected into a C18 250 × 4.6 mm, 5 μm Jupiter analytical column (Phenomenex, Torrance, CA) on the same ÄKTA HPLC system and with the same eluents. The gradient was adjusted to isocratic 30% eluent B for 5 minutes, and then increased to 100% B during 120 minutes.

### Antimicrobial assays

Assays for antimicrobial activity and MIC assays were carried out as reported earlier against *Escherichia coli* ATCC 25922, *Staphylococcus aureus* ATCC 29213, *Pseudomonas aeruginosa* ATCC27853, and *Candida albicans* ATCC 90028 [11], or according to international standardized protocol [47].

### Structure determination of isolated compound

The fractions that showed antimicrobial activity were analyzed with a Nano Acquity UPLC system coupled to a quadropole time-of-flight mass spectrometer (Qtof Micro, Waters, Milford, MA). During MS analysis the capillary voltage was 4100 V, sample cone 30 V, and the collision energy was set to 5 V, but increased to 15, 25, 40, 55, and 65 V with a 1 second interscan time during MS/MS analysis. The column was a C18 Nano Acquity 75 μm × 250 mm column with 1.7 μm particle size (Waters, Milford, MA). The lock mass was a 1.27 μM solution of (Glu^1^)-Fibrinopeptide B (Sigma Aldrich, St Louis, MO), infused at a flow rate of 0.4 μL/min. All spectra and chromatograms were analyzed with MassLynx version 4.0 software package. The two eluents: A and B, were water and acetonitrile, respectively, with 0.1% formic acid at a flow rate of 0.250 μL/min. All samples were injected and separated on the column with an isocratic step of 1% B for 1 minute, and a gradient increasing to 90% B during 49 minutes, and held for 4 minutes. To avoid possible microbial growth in eluent A, 0.05% MeOH was added.

^1^H NMR experiments were performed on a Bruker 600 MHz spectrometer equipped with a smartprobe, using tetramethylsilane as internal standard. All samples were dissolved in 99.9% dimethylsulfoxide-*d6* and analyzed at 25°C.

## Availability of supporting data

This article contains no supporting data.
